# Identification of CXCL16 as a diagnostic biomarker for obesity and intervertebral disc degeneration based on machine learning

**DOI:** 10.1038/s41598-023-48580-w

**Published:** 2023-12-03

**Authors:** Jiahao Liu, Jian Zhang, Xiaokun Zhao, Chongzhi Pan, Yuchi Liu, Shengzhong Luo, Xinxin Miao, Tianlong Wu, Xigao Cheng

**Affiliations:** 1 Department of Orthopedics, The Second Affiliated Hospital of Nanchang University, Nanchang, 330006 Jiangxi China; 2 Institute of Orthopedics of Jiangxi Province, Nanchang, 330006 Jiangxi China; 3 Institute of Minimally Invasive Orthopedics, Nanchang University, Nanchang, 330006 Jiangxi China

**Keywords:** Computational biology and bioinformatics, Molecular biology

## Abstract

Intervertebral disc degeneration (IDD) is the primary cause of neck and back pain. Obesity has been established as a significant risk factor for IDD. The objective of this study was to explore the molecular mechanisms affecting obesity and IDD by identifying the overlapping crosstalk genes associated with both conditions. The identification of specific diagnostic biomarkers for obesity and IDD would have crucial clinical implications. We obtained gene expression profiles of GSE70362 and GSE152991 from the Gene Expression Omnibus, followed by their analysis using two machine learning algorithms, least absolute shrinkage and selection operator and support vector machine-recursive feature elimination, which enabled the identification of C-X-C motif chemokine ligand 16 (CXCL16) as a shared diagnostic biomarker for obesity and IDD. Additionally, gene set variant analysis was used to explore the potential mechanism of CXCL16 in these diseases, and CXCL16 was found to affect IDD through its effect on fatty acid metabolism. Furthermore, correlation analysis between CXCL16 and immune cells demonstrated that CXCL16 negatively regulated T helper 17 cells to promote IDD. Finally, independent external datasets (GSE124272 and GSE59034) were used to verify the diagnostic efficacy of CXCL16. In conclusion, a common diagnostic biomarker for obesity and IDD, CXCL16, was identified using a machine learning algorithm. This study provides a new perspective for exploring the possible mechanisms by which obesity impacts the development of IDD.

## Introduction

Intervertebral disc degeneration (IDD) onstitutes a significant aetiology of lumbar and cervical spinal discomfort^1^. The occurrence of IDD leads to a series of diseases in the spine, resulting in a significant burden on the global health care system^2,3^. The predominant surgical techniques currently employed involve excision of symptomatic intervertebral discs, which can result in compromised functionality, immobilization, and potential complications arising from altered biomechanics^4^. The pathogenesis of IDD remains unclear at present^5^. Considering the complexity of and variability in the factors leading to IDD, it is imperative to identify various triggers and discover distinct diagnostic biomarkers that are aetiology-specific. Such biomarkers could facilitate more precise prevention and treatment of the populations afflicted with IDD.

The risk of IDD is determined by a multitude of factors, as revealed by previous studies^6–8^. It has been postulated that there may exist a potential risk factor linking IDD to obesity^9,10^. An upwards trend has been noticed in the incidence of obesity-related low back pain and IDD^11^. A recent review also pointed out that obesity plays an important role in all diseases that may lead to the occurrence and persistence of low back pain in children^12^. Furthermore, it is important to note that in adolescent patients, a significant association has been observed between body mass index (BMI) and IDD, with overweight and obese patients demonstrating greater severity of IDD compared to normal-weight patients^13^. Additionally, according to a comprehensive meta-analysis, one of the primary risk factors for IDD is obesity^14^. Moreover, it has been reported that IDD may be influenced by obesity through direct biochemical effects of fatty acids on intervertebral disc cell metabolism^15^. The healing process may be impaired due to the nutritional interference of intervertebral discs caused by obesity^16^. Additionally, functional leptin receptor expression in intervertebral disc tissue suggests the potential regulation of cell functions by leptin^17^. The possible biochemical connection between IDD and obesity is supported by these collective findings. However, common diagnostic biomarkers for IDD and obesity have yet to be reported. Identifying common diagnostic biomarkers for obesity and IDD would greatly aid in the prevention and treatment of IDD in obese patients, thus representing considerable clinical value.

The challenge of biomarker discovery lies in achieving effective feature selection, a fundamental task in this field^18,19^. To address this issue, a combination of several machine learning approaches can be utilized^20^. In recent years, the application of advanced machine learning algorithms has become prevalent in the screening of medical biomarkers ^21,22^. The least absolute shrinkage and selection operator (LASSO), which combines feature selection and regularization, is a machine learning technique that can construct a penalty function to address complex collinearity data and produce an improved linear model. The refined linear model generated by the lasso algorithm is an effective approach to solving this problem^23^. Similarly, the extensively utilized machine learning technique support vector machine-recursive feature elimination (SVM-RFE) finds wide application in various biological fields characterized by a feature size that surpasses the available number of samples, as observed in metabolomic analyses^24^. The aim of this study was to screen for shared diagnostic biomarkers associated with obesity and IDD using two machine algorithms, LASSO and SVM-RFE, with the ultimate goal of identifying potential therapeutic and diagnostic targets for the management and prevention of obesity-related IDD.

## Materials and methods

### Dataset download and data preprocessing

The raw gene expression profile data were obtained from the Gene Expression Omnibus (GEO) by conducting a search for RNA-seq profiles using relevant keywords such as "obesity" and "intervertebral disc degeneration". Two training datasets (GSE70362 and GSE152991) and two validation datasets (GSE124272 and GSE59034) were identified. GSE70362 comprised 16 IDD subjects and 8 normal controls, while GSE152991 included 34 obese patients and 11 normal controls. The validation datasets consisted of GSE124272 (8 IDD patients and 8 controls) and GSE59034 (16 obese patients and 16 normal controls). The data were subjected to background correction and normalization using the R package "limma".

### Identification of differentially expressed genes (DEGs)

The identification of DEGs between samples from healthy controls and patients was conducted utilizing the R package "limma" with a screening criterion consisting of a Wilcoxon test and a *p* value threshold of less than 0.05.

### Construction and module analysis via weighted gene coexpression network analysis (WGCNA)

The WGCNA software package R.4.2.3, which employs weighted gene coexpression network analysis, was utilized to conduct coexpression network construction and correlation analysis between clinical features regarding obesity and IDD. Key modules associated with the diseases were identified based on the eigengenes' Pearson correlation coefficient and *P* value.

### GO and KEGG enrichment analysis of shared genes

Correlation analysis among DEGs was performed using the R package "corrplot". To explore comprehensive information on large-scale gene data, GO enrichment analysis and KEGG pathway enrichment analysis^25^ (https://www.kegg.jp) were employed as common bioinformatic methods. The results of these analyses were visualized using the "GOplot" program package. In addition, Metascape (http://metascape.org), an online analytical tool, was utilized to analyse possible diseases (DisGeNET database) related to shared genes.

### Screening diagnostic biomarkers via machine learning algorithms

Diagnostic biomarkers for obesity and IDD were screened using two machine learning algorithms: LASSO and SVM-RFE. The R packages "glmnet" and "e1071" were utilized to perform LASSO regression and SVM-RFE. A shared diagnostic biomarker for the two aforementioned diseases was obtained by employing a Venn plot.

### Development and verification of a diagnostic biomarker

The significance of the difference in diagnostic biomarkers between the disease and control groups was evaluated using a significance threshold of *p* < 0.05, with subsequent assessment of the potential diagnostic biomarker's predictive ability in the validation dataset carried out through receiver operating characteristic (ROC) analysis utilizing the "pROC" package.

### Gene set variation analysis (GSVA) of the diagnostic biomarker

To investigate the potential biological functions of the diagnostic biomarker, GSVA was conducted using the R package "clusterprofiler" based on GO and KEGG gene sets. The reference gene sets c5.go.Symbols.gmt and c2.cp.kegg.Symbols.gmt were employed. Enriched functional categories and pathways were identified by applying a cut-off criterion of a p-adjusted value of < 0.05.

### Evaluation and correlation analysis of immune infiltrating cells

The extent of immune cell infiltration and the nature of infiltrating immune cells were explored through single-sample gene set enrichment analysis (ssGSEA) of 28 immune-related signatures derived from expression profiles to reveal differences in the correlations between the diagnostic biomarker and immune infiltrating cells. Using "gsva" function for ssGSEA. Furthermore, the "ggplot2" package was employed to visualize the results.

### Statistical analysis

All data processing and analysis were performed using R software (version 4.2.3). Differences between independent and non-normally distributed variables were analyzed using the Wilcoxon test. Spearman correlation analysis was conducted to calculate the correlation coefficients between different genes, the correlation between genes and immune cells, as well as the module-trait associations. *P* < 0.05 was considered statistically significant.

## Results

### Identification of shared genes in IDD and obesity

**Figure 1:**
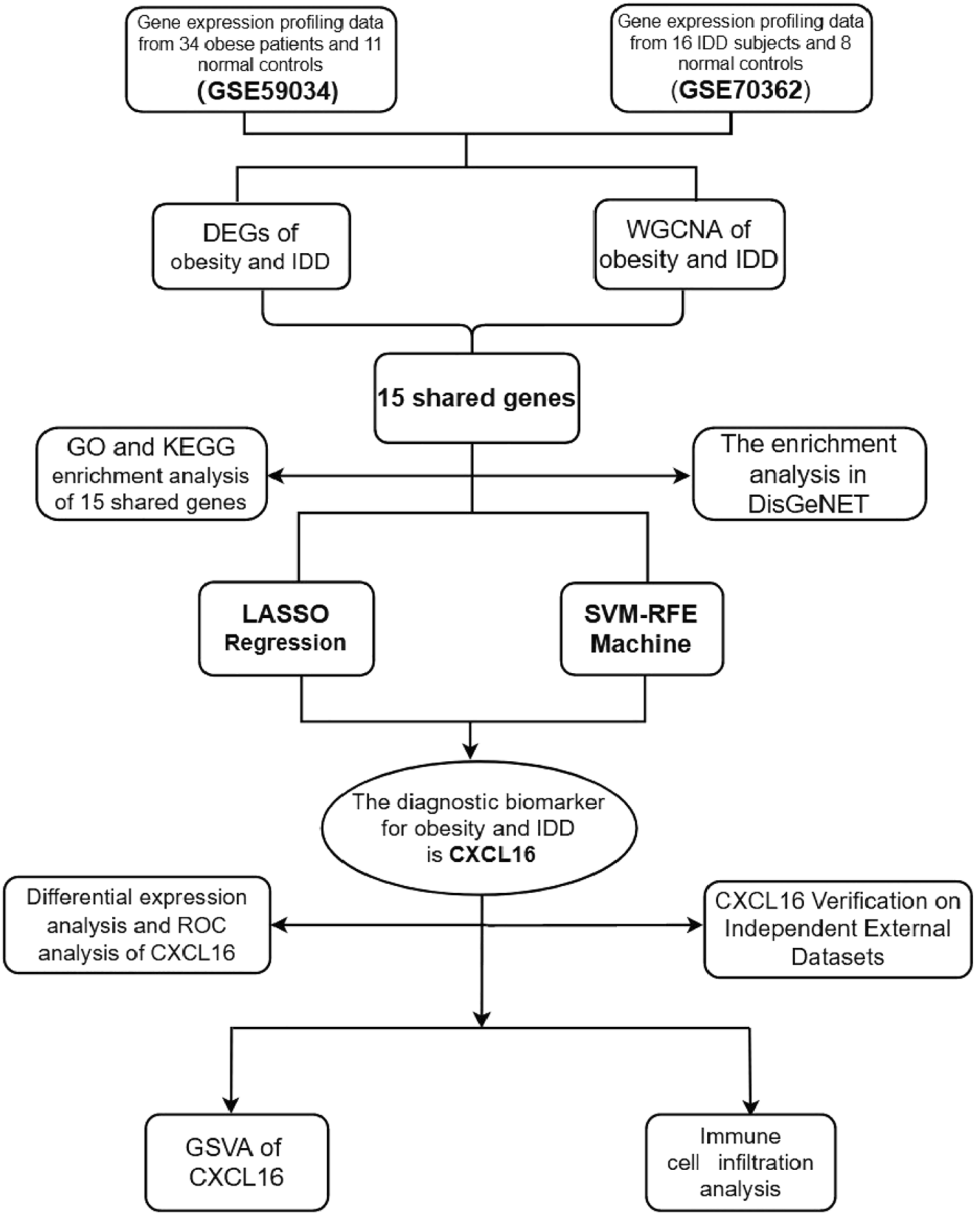
Analysis flow chart.

**Figure 2:**
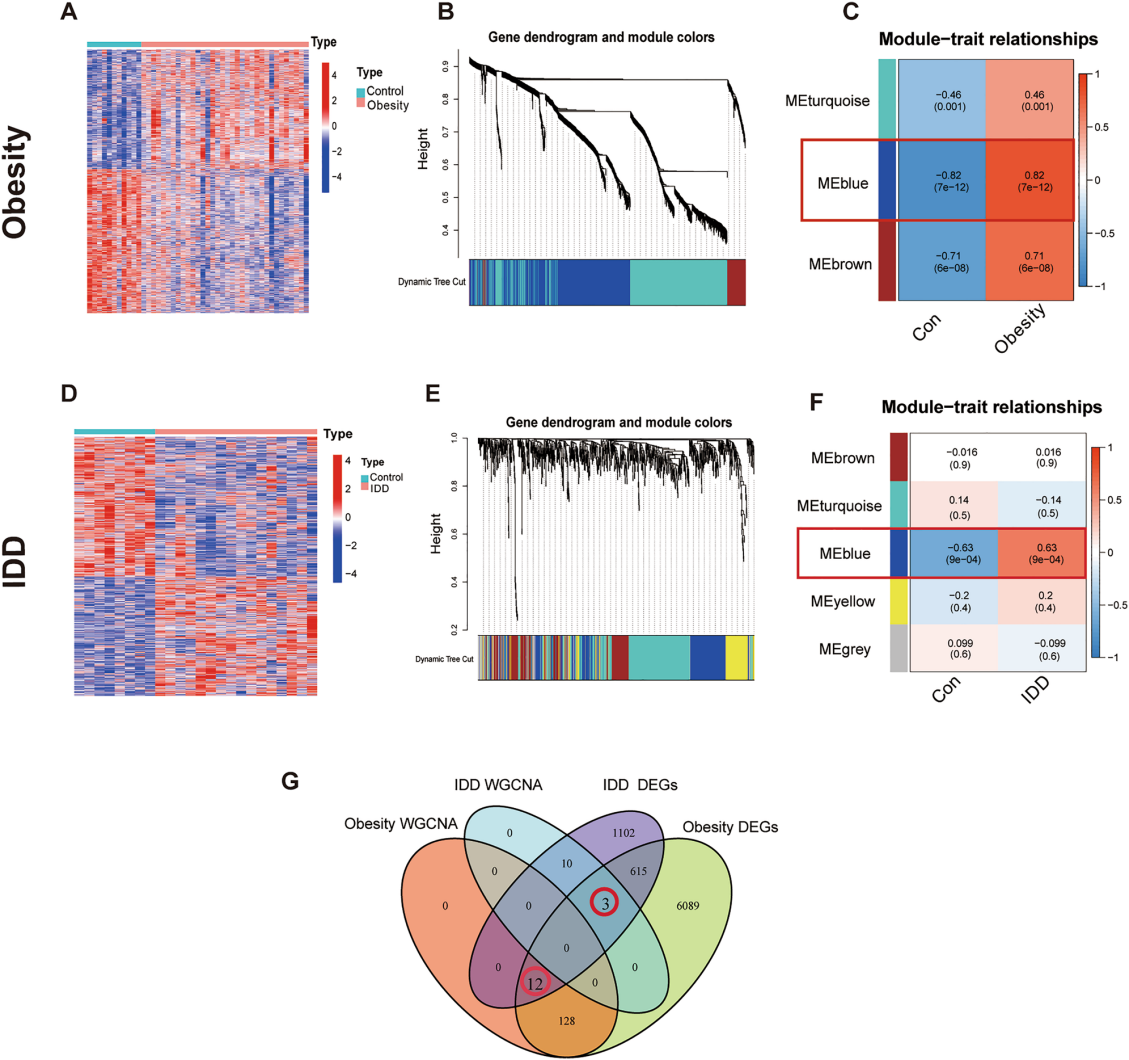
DEGs and WGCNA of obesity and IDD. (**A**) Heatmap plots of DEGs in obesity. (**B**) The cluster dendrogram of coexpression in obesity. (**C**) Correlation between modules and clinical training in obesity. (**D**) Heatmap plots of DEGs of IDD. (**E**) The cluster dendrogram of coexpression in IDD. (**F**) Correlation between modules and clinical training in IDD. (**G**) The Venn diagram shows an overlap of 15 genes according to DEG analysis and WGCNA between IDD and obesity.

To clearly demonstrate the specific process of this study, the bioinformatics analysis process is summarized in Fig. 1**.** A total of 3102 upregulated and 3744 downregulated DEGs were detected between obese patients and normal controls (Fig. 2A)**.** In obesity, the relationships between modules were evaluated by drawing a heatmap based on the Spearman correlation coefficient for the module-trait associations (Fig. 2B). Three modules for obesity were identified by WGCNA, and the blue module was clinically most significant and was chosen as an obesity-related module (r = − 0.82, *p* = 7e−12, genes = 140) (Fig. 2C). Additionally, a total of 1741 DEGs were identified between IDD patients and normal controls, of which 802 DEGs were upregulated and 939 DEGs were downregulated, as revealed in the heatmaps (Fig. 2D). In IDD, the relationships between modules were evaluated by drawing a heatmap based on the Spearman correlation coefficient for the module-trait associations (Fig. 2E). Five modules for IDD were identified by WGCNA, and the ME in the blue module was clinically more significant for IDD than any other module, which was chosen as the IDD-related module (r = − 0.63, *p* = 9e−04, genes = 13) (Fig. 2F). By intersecting genes in DEGs and the most positive modules, a total of 15 shared genes were obtained, which may be associated with the pathogenesis of obesity and IDD (Fig. 2G).

### Enrichment of functions and pathways of shared genes

**Figure 3:**
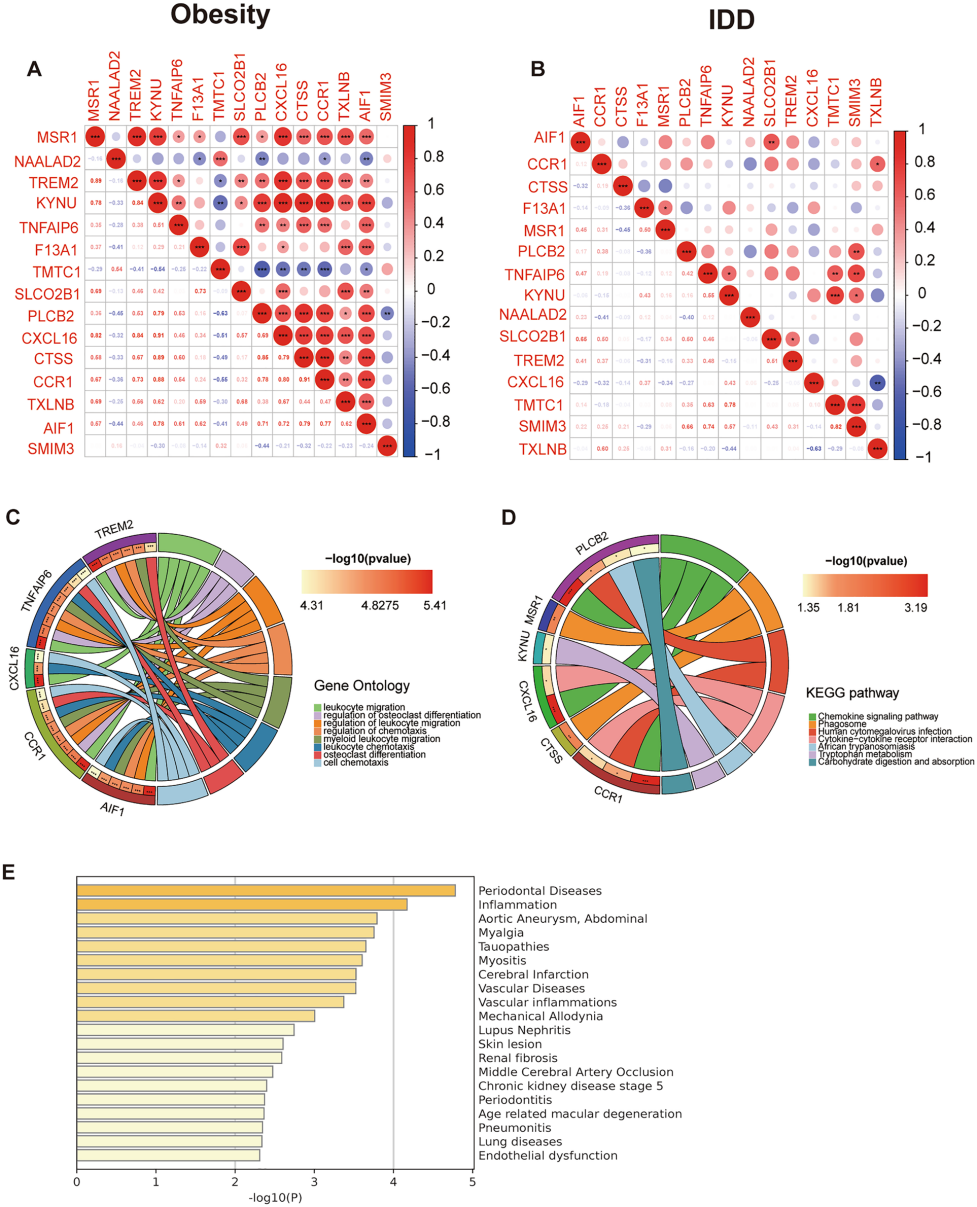
Enrichment analysis of 15 shared genes. (**A**) Correlation analysis of 15 shared genes for obesity. (**B**) Correlation analysis of 15 shared genes for IDD. (**C**) GO analysis of 15 shared genes. (**D**) KEGG analysis of 15 shared genes for obesity. (**E**) Summary of enrichment analysis in DisGeNET.

A strong and significant correlation was observed between most shared genes in the obesity samples (Fig. 3A), as was the case in the IDD samples (Fig. 3B). The GO analysis showed that shared genes were mainly enriched in the regulation of osteoclast differentiation, leukocytes and chemotaxis (Fig. 3C). Additionally, KEGG analysis revealed a significant enrichment in shared genes in the chemokine signalling pathway, cytokine‒cytokine receptor interaction, and carbohydrate digestion and absorption (Fig. 3D). The enrichment analysis in DisGeNET showed that shared genes were associated with inflammation and myalgia (Fig. 3E).

### Identification of potential shared diagnostic markers

**Figure 4:**
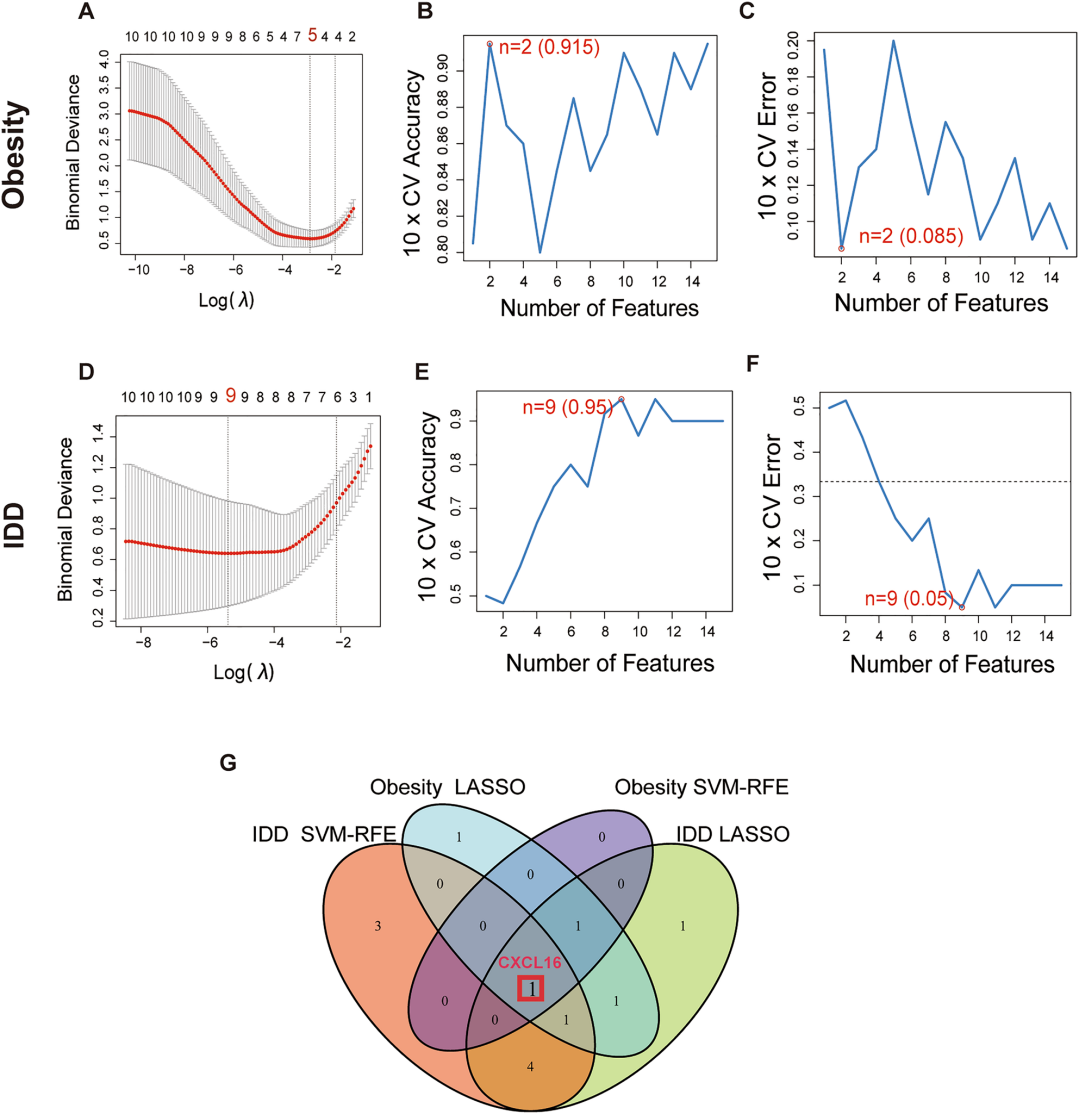
Machine learning to screen diagnostic markers. (**A**) LASSO to screen diagnostic markers for obesity. (**B**, **C**) SVM-RFE to screen diagnostic markers for obesity. (**D**) LASSO to screen diagnostic markers for IDD. (**E**, **F**) SVM-RFE to screen diagnostic markers for IDD. (**E**) Venn diagram showing a diagnostic gene in IDD and obesity.

LASSO and SVM-RFE are both machine learning methods for screening characteristic genes. Based on the 15 shared genes, 5 possible diagnostic biomarkers for obesity were identified by LASSO (Fig. 4A), and 2 possible diagnostic biomarkers for obesity were identified by SVM-RFE (Fig. 4B, C) Additionally, 9 possible diagnostic biomarkers for IDD were identified by LASSO (Fig. 4D), and 9 possible diagnostic biomarkers for IDD were identified by SVM-RFE (Fig. 4E, F). Ultimately, we determined that C-X-C motif chemokine ligand 16 (CXCL16) was the best diagnostic biomarker for the development of IDD and obesity by Venn analysis (Fig. 4G).

### Differential expression analysis and ROC curve of CXCL16 in obesity and IDD

**Figure 5:**
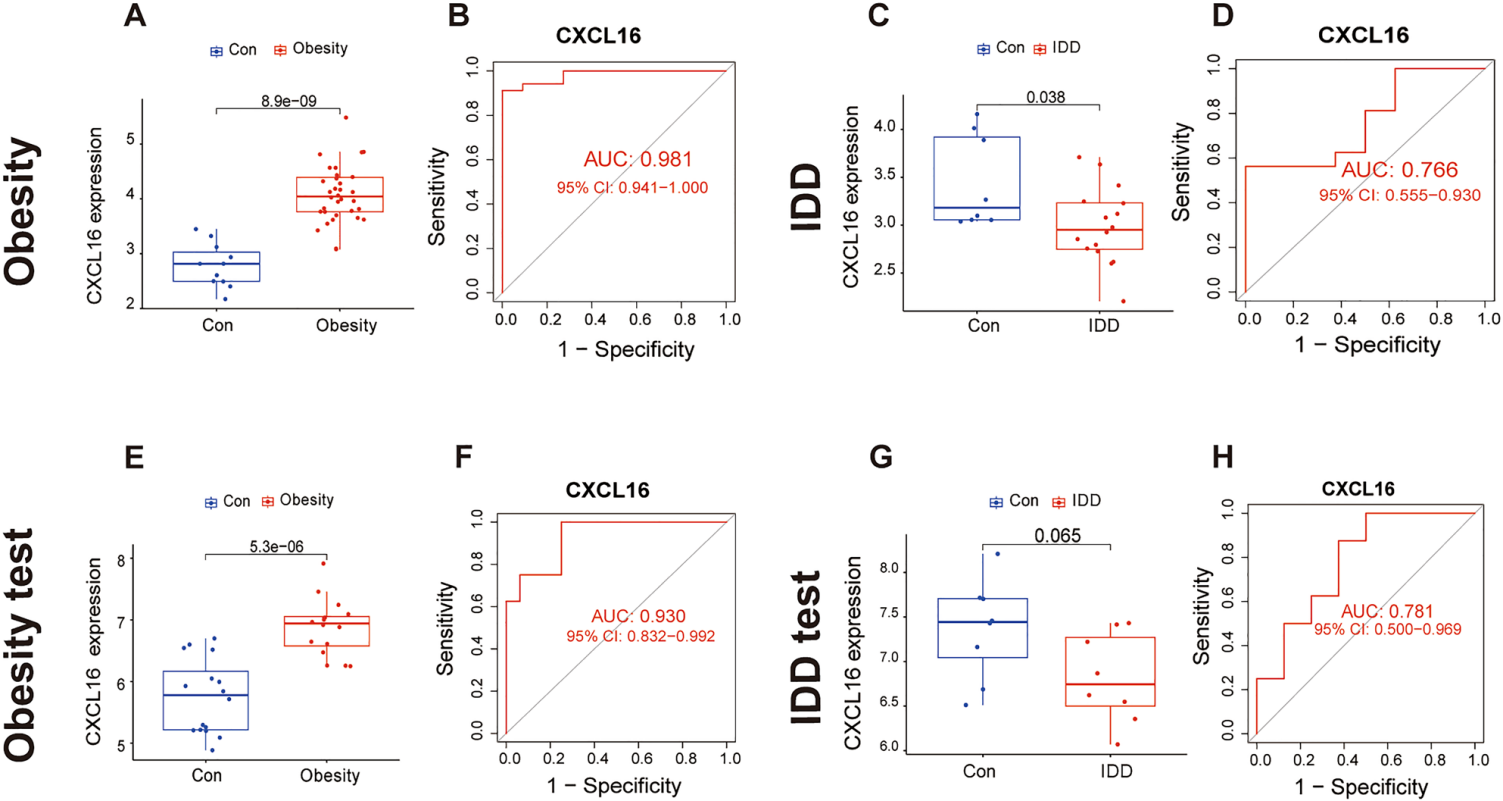
Differential expression analysis and ROC curve of CXCL16. (**A**) Differential expression of CXCL16 in obesity. (**B**) ROC curve evaluating the CXCL16 diagnostic efficacy in obesity. (C) Differential expression of CXCL16 in IDD. (**D**) ROC curve evaluating the CXCL16 diagnostic efficacy in IDD. (**E**) Differential expression of CXCL16 in the obesity test cohort. (**B**) ROC curve evaluating the CXCL16 diagnostic efficacy in the obesity test cohort. (**C**) Differential expression of CXCL16 in the IDD test cohort. (**D**) ROC curve evaluating the CXCL16 diagnostic efficacy in the IDD test cohort.

We found that CXCL16 was significantly differentially expressed between the disease group and the control group and had good diagnostic efficacy according to the ROC curve. Differential expression analysis showed that CXCL16 was significantly overexpressed in obesity (Fig. 5A) and significantly underexpressed in IDD (Fig. 5C). By ROC curve analysis, we obtained an AUC of 0.981 for obesity (Fig. 5B), and the results showed that the AUC was 0.766 for IDD (Fig. 5D)**.** The same results for CXCL16 were verified in an independent obesity validation dataset, GSE59034 (Fig. 5E), and an independent IDD validation dataset, GSE124272 (Fig. 5G). The ROC curve showed an AUC = 0.930 in the obesity test cohort (Fig. 5F) and an AUC = 0.781 in the IDD test cohort (Fig. 5H).

### GSVA enrichment analysis of CXCL16 in IDD and obesity

**Figure 6:**
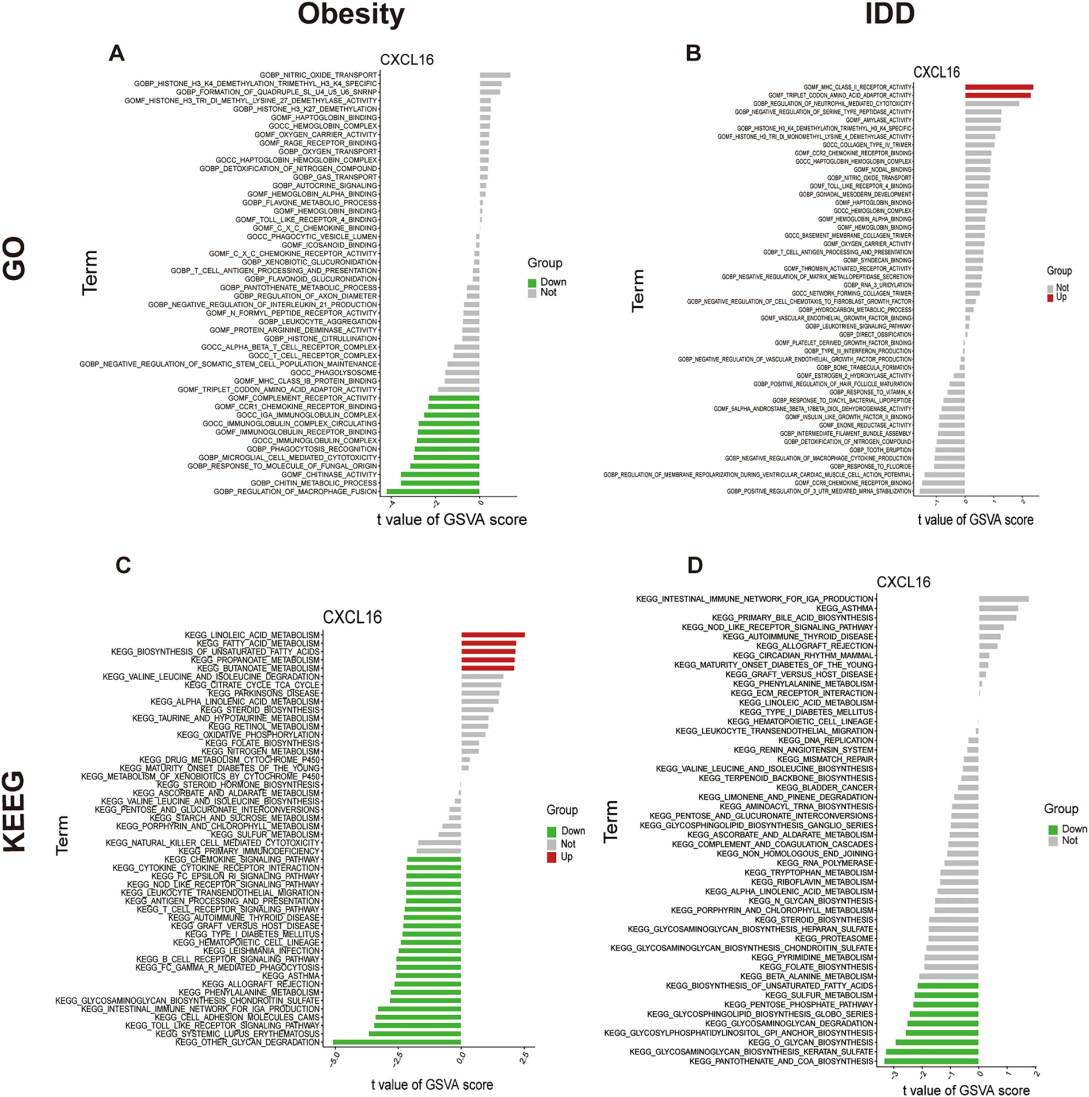
GSVA of CXCL16. (**A**) GO analysis based on GSVA of CXCL16 in obesity. (**B**) GO analysis based on GSVA of CXCL16 in IDD. (**C**) KEGG analysis based on GSVA of CXCL16 in obesity. (**D**) KEGG analysis based on GSVA of CXCL16 in IDD.

In obesity, the GO functions of the CXCL16 low-expression group were enriched in the regulation of macrophage fusion and chitin metabolic processes (Fig. 6A). The KEGG pathways of the CXCL16 high-expression group were mainly enriched in linoleic acid metabolism and fatty acid metabolism, and in the CXCL16 low-expression group of obesity, the pathways were mainly enriched in other glycan degradation and toll-like receptor signalling pathways (Fig. 6C). In IDD, GO functions of the CXCL16 high-expression group were enriched in mhc-class-ii-receptor-activity and triple codon amino acid adaptor activity (Fig. 6B). KEGG pathways of the CXCL16 low-expression group were mainly enriched in pantothenate and CoA biosynthesis and glycosaminoglycan, specifically keratan sulfate, biosynthesis (Fig. 6D).

### Immune cell infiltration analysis

**Figure 7:**
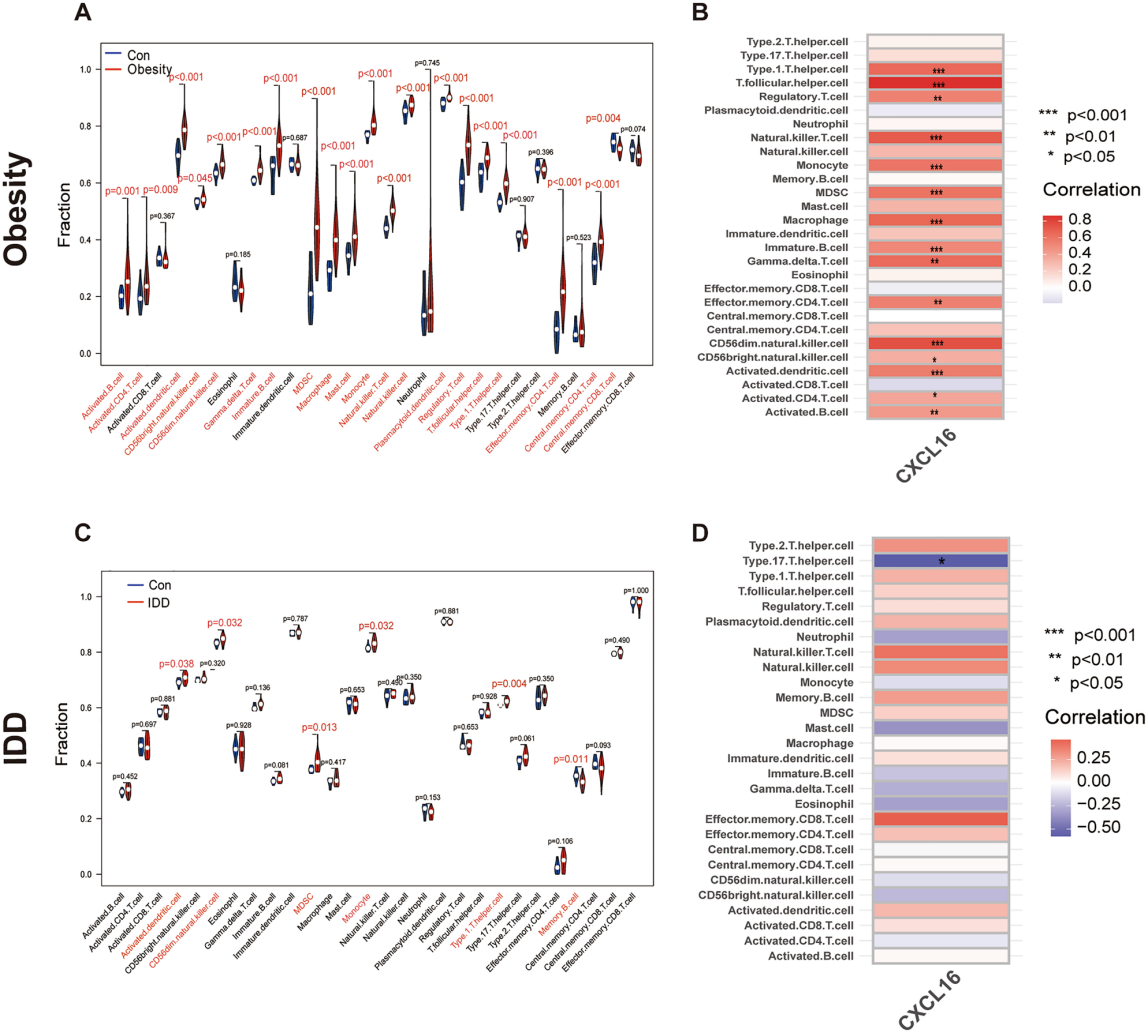
Immune cell infiltration analysis. (**A**) Immune cell infiltration analysis in GSE152991. (**B**) Correlation analysis between CXCL16 and immune cell infiltration in obesity. (**C**) Immune cell infiltration analysis in GSE70362. (**D**) Correlation analysis between CXCL16 and immune cell infiltration in IDD.

The effect of immune cells on disease was analysed by comparing differences in immune cell levels between the normal and control groups in the 2 datasets using ssGSEA. In GSE152991, lower levels of central memory CD8 T cells were observed in obesity samples compared to normal controls. Levels of activated B cells, activated CD4 T cells, activated dendritic cells, CD56 bright natural killer cells, CD56dim natural killer cells, gamma delta T cells, immature B cells, myeloid-derived suppressor cells (MDSC), macrophages, mast cells, monocytes, natural killer T cells, natural killer cells, plasmacytoid dendritic cells, regulatory T cells, T-follicular helper cells, type 1 T helper cells, effector memory CD4 T cells and central memory CD4 T cells were higher in obesity samples than in normal control samples (Fig. 7A). In GSE70362, lower levels of memory B cells were found in IDD sample tissues compared to normal tissues. In addition, activated dendritic cells, CD56dim natural killer cells, MDSC, monocytes and type 1 T helper cell levels were higher in IDD samples than in normal control samples (Fig. 7C). In obesity, CXCL16 positively regulates the following immune cell infiltrations as shown by correlation analysis between diagnostic biomarkers and immune cells: type 1 T helper cells, T-follicular helper cells, regulatory T cells, natural killer T cells, monocytes, MDSC, macrophages, immature B cells, gammadelta T cells, effector memory CD4 T cells, CD56dim natural killer cells, activated dendritic cells, activated CD4 T cells and activated B cells (Fig. 7B). In IDD, CXCL16 negatively regulates type 17 T-helper cell infiltration (Fig. 7D).

## Discussion

IDD is negatively affected by obesity, and recent studies suggest that adiposity, rather than excess body mass, is responsible for this detrimental effect^26^. The pathological process of IDD involves various factors, among which obesity is implicated as a risk factor contributing to mechanical stress and inflammation^10^. Therefore, obesity may be a potential target for the treatment of IDD in the near future^27^. Despite numerous studies, including basic experiments and clinical cross-sectional investigations, the mechanism underlying the association between obesity and disc degeneration remains unrecognized^28^. In this study, a common diagnostic biomarker for IDD and obesity, CXCL16, was identified with machine learning algorithms, and the potential mechanism of CXCL16 action was explored by GSVA. Furthermore, we analysed the regulatory effect of CXCL16 on immune cell infiltration, and our findings may provide potential applications for obese and IDD patients, such as early diagnosis, treatment monitoring.

The CXC chemokine subfamily encompasses various members, one of which is CXCL16. In contrast to other members of this subgroup, CXCL16 possesses a unique structural composition consisting of four distinct domains. A chemokine domain is present, which is connected to the cell surface through a mucin-like stack, and this stack is further linked to transmembrane and cytoplasmic domains^29^. It plays a significant role in the progression of cancer^30–32^ as well as the course of atherosclerosis, renal fibrosis, nonalcoholic fatty liver disease (NAFLD) and pulmonary fibrosis^33–38^. The promotion of disc degeneration by CXCL16 was found to affect fatty acid metabolism, which is consistent with previous research. Previous studies have demonstrated that disk cell metabolism is directly influenced by fatty acids in the context of obesity-mediated apoptosis and extracellular matrix metabolic imbalances via MAPK pathway activation in intervertebral disk degeneration^15^. However, previous studies have not identified the specific molecular mechanism by which nucleus pulposus cell apoptosis occurs through the MAPK pathway, and the diagnostic biomarker we have selected this time, CXCL16, is likely to be involved in the MAPK pathway as a potential biomolecule that promotes IDD.

By analysing the correlation between CXCL16 and immune cells, we found that CXCL16 negatively regulates T helper 17 cells (TH17 cells). Interleukin 17-producing TH17 cells, which belong to a subtype of helper T cells, are recognized as crucial drivers in the development of both autoimmunity and inflammation^39^. TH17 cells of human origin are frequently observed in peripheral tissues and organs ^40,41^. Numerous human diseases may be influenced by TH17-cell activity and should not be overlooked ^42–44^. During an inflammatory response, the primary role of TH17 cells is to facilitate the recruitment of inflammatory immune cells to infected or damaged tissue. This can result in the exacerbation of autoimmune diseases and chronic inflammatory conditions^45^. On the basis of our analysis, we found that in IDD, CXCL16 expression was low, which promoted the infiltration of TH17 cells in the intervertebral disc, which may exacerbate the inflammatory response of the intervertebral disc and thus promote degeneration of the intervertebral disc.

Although diagnostic biomarkers associated with obesity and IDD have been identified by machine learning algorithms, their diagnostic efficiency has been confirmed in external datasets. However, this study only showed the correlation of CXCL16 in the effects of IDD and obesity, and whether CXCL16 mediates the development of IDD in obese patients needs to be further investigated. In addition, our study is limited to basic sample characteristics and expression data obtained from GEO database. We were unable to access more detailed clinical information, such as age, complications, BMI, degree of IDD by the Pfirrmann grade, involved disc levels, and type of IDD (e.g., disc herniation, spinal stenosis, degenerative spondylolisthesis, disc disease), which restricts our ability to rule out other confounding factors that may affect the expression of CXCL16 and other inflammatory marker genes. This limitation in our study can potentially influence the interpretation of the identified common diagnostic markers from both IDD and obesity-related gene expression datasets. In future studies, we will validate our conclusions by designing prospective cohort studies to collect more comprehensive and multidimensional data. Moreover further experimental validation is needed to comprehensively understand the role of this diagnostic marker and its potential regulatory mechanisms related to IDD and obesity by techniques such as RT‒qPCR and Western blotting.

## Conclusions

We identified CXCL16 as a diagnostic biomarker for obesity and IDD in this study by machine learning methods. CXCL16 was investigated by GSVA to promote disc degeneration in IDD mainly by affecting fatty acid metabolism, as demonstrated by diagnostic biomarker and immune cell correlation analysis, revealing that CXCL16 promotes disc degeneration by regulating T helper 17 cell infiltration. Our analysis provides a new perspective for further exploring the underlying mechanisms by which obesity impacts IDD, and we will subsequently perform relevant in vitro and in vivo experiments.

## Data Availability

The datasets analyzed in this study can be obtained from the NCBI repository using the accession numbers GSE152991, GSE70362, GSE124272, and GSE59034.

## Abbreviations

IDD: Intervertebral disc degeneration
BMI: Body mass index
LASSO: Least absolute shrinkage and selection operator
SVM-RFE: Support vector machine-recursive feature elimination
DEGs: Differentially expressed genes
GEO: Gene Expression Omnibus
WGCNA: Weighted gene coexpression network analysis
ROC: Receiver operating characteristic
GSVA: Gene set variation analysis
ssGSEA: Single-sample gene set enrichment analysis
CXCL16: C-X-C motif chemokine ligand 16
MDSC: Myeloid-derived suppressor cells
TH17 cells: T helper 17 cells
